# Surface Modification of Additively Manufactured Nitinol by Wet Chemical Etching

**DOI:** 10.3390/ma14247683

**Published:** 2021-12-13

**Authors:** Denis Nazarov, Aida Rudakova, Evgenii Borisov, Anatoliy Popovich

**Affiliations:** 1 Institute of Machinery, Materials and Transport, Peter the Great Saint Petersburg Polytechnic University, Polytechnicheskaya, 29, 195221 Saint Petersburg, Russia; evgenii.v.borisov@gmail.com (E.B.); director@immet.spbstu.ru (A.P.); 2 Research Centre “Innovative Technologies of Composite Nanomaterials”, Saint Petersburg State University, Universitetskaya Nab, 7/9, 199034 Saint Petersburg, Russia; 3 Laboratory “Photoactive Nanocomposite Materials”, Saint Petersburg State University, Ulianovskaia Str. 1, Peterhof, 198504 Saint Petersburg, Russia; aida.rudakova@spbu.ru

**Keywords:** additive manufacturing, nitinol, chemical etching, surfaces, wettability, biomaterials

## Abstract

Three-dimensional printed nitinol (NiTi) alloys have broad prospects for application in medicine due to their unique mechanical properties (shape memory effect and superplasticity) and the possibilities of additive technologies. However, in addition to mechanical properties, specific physicochemical characteristics of the surface are necessary for successful medical applications. In this work, a comparative study of additively manufactured (AM) NiTi samples etched in H_2_SO_4_/H_2_O_2_, HCl/H_2_SO_4,_ and NH_4_OH/H_2_O_2_ mixtures was performed. The morphology, topography, wettability, free surface energy, and chemical composition of the surface were studied in detail. It was found that etching in H_2_SO_4_/H_2_O_2_ practically does not change the surface morphology, while HCl/H_2_SO_4_ treatment leads to the formation of a developed morphology and topography. In addition, exposure of nitinol to H_2_SO_4_/H_2_O_2_ and HCl/H_2_SO_4_ contaminated its surface with sulfur and made the surface wettability unstable in air. Etching in NH_4_OH/H_2_O_2_ results in surface cracking and formation of flat plates (10–20 microns) due to the dissolution of titanium, but clearly increases the hydrophilicity of the surface (values of water contact angles are 32–58°). The etch duration (30 min or 120 min) significantly affects the morphology, topography, wettability and free surface energy for the HCl/H_2_SO_4_ and NH_4_OH/H_2_O_2_ etched samples, but has almost no effect on surface composition.

## 1. Introduction

Nearly equiatomic nickel–titanium (NiTi) alloys are used in many biomedical applications (stents, dental braces, surgical instruments, implants, etc.) due to their unique shape memory effect, superelasticity, relatively low modulus of elasticity, as well as good biocompatibility [1,2]. The rapid and successful development of 3D printing in recent years opens up even broader prospects for the use of NiTi in personalized medicine [1,3]. However, in addition to mechanical properties and specialized design, the biomaterial must have a number of specific characteristics such as morphology, topography, charge, and surface chemical composition [4]. Depending on the application, the material must be either bioinert or bioactive. At the same time, there are many requirements for materials resistance to biocorrosion, antibacterial properties, interaction with living cells, tissues, and the immune system [5]. In order for the material to meet such a set of characteristics, it is necessary to modify its surface. 

Many surface modification methods are applied to nitinol. Micromachining methods including water jet machining (WJM), laser surface texturing (LST), electrochemical machining, and Micro-Electrical Discharge Machining (Micro-EDM) are actively used to remove material from the surface and produce developed topography [6]. WJM is promising since modified surfaces are not subjected to heat-related processes, and there are no formed cracks or deformed structures [7]. However, there are challenges relating to the depth homogeneity due to the strain hardening and yield strength [6]. LST is a thermal process to remove the material without any mechanical contact between NiTi and the laser beam. Therefore LST does not depend on the mechanical properties of the material. This method is very precise and allows you to adjust the surface topography in a wide range, but at the same time, it is quite expensive for scaling and industrial applications [8,9]. Electrochemical methods including anodization are also independent of the mechanical properties of NiTi and allow produce and precisely regulate topography and morphology of the surface [9,10]. However, these methods, as well as plasma treatment and ion implantation, are energy consuming and additional treatments are usually required to further improving medical properties [11].

Wet chemical etching (CE) is an effective and technically very simple method of forming surfaces with developed micro- and nanoscale morphology and modifying the surface composition by varying the nature of the etching agent, its composition, etching time and temperature [5,12]. CE of pure titanium and many of its medical alloys is very well studied and intensively used in industry [13,14]. For example, chemical etching using an H_2_SO_4_/HCl mixture is part of sandblasting and acid etching (SLA) technique to create a developed topography and hydrophilic surface, that accelerates and improves the osseointegration of titanium implants [15]. Despite this, the etching of nitinol is still very poorly studied, and the features of this process for 3D-printed NiTi samples are not known at all. To date, there are data on the CE of NiTi in acidic mixtures HF/HNO_3_/H_2_O [16,17], HCl/HNO_3_ [18] two-stage treatment using HCl/HF/H_3_PO_4_ and HNO_3_/HCl mixtures [19], and its surface modification with NaOH [20] and sequential exposure to NaOH and HNO_3_ [21]. The use of etchants of complex composition, as well as multi-step processes, allows for creating a complex hierarchical morphology, which is extremely important for nitinol applications as implants [4,22]. However, to the best of our knowledge, no data on the peculiarities of the nitinol etching process exist yet.

In this work, the effect of etchant composition and etching time on the physicochemical properties of the surface of nitinol produced by selective laser melting (SLM) was studied. Three types of etchants were chosen, the H_2_SO_4_/HCl mixture used in SLA technology [15], and piranha solutions (H_2_SO_4_/H_2_O_2_ and NH_4_OH/H_2_O_2_), which were successfully used for etching the titanium surface previously [23,24,25].

## 2. Materials and Methods

### 2.1. Samples Preparation

In our study, nitinol rods were obtained by selective laser melting (SLM). Commercial powders of nearly equiatomic NiTi alloys (CNPC Powder, Shanghai, China) were gas atomized. Samples were prepared using an SLM 280HL selective laser melting machine (SLM Solutions GmbH, Luebeck, Germany). The machine is equipped with two lasers with a power of 400 W and 1 kW [26].

Before etching, the NiTi rods (13 mm diameter, 40 cm length) were cut into discs (2–3 mm thick) using a Buehler IsoMet 1000 (Buehler, Lake Bluff, IL, USA). The obtained discs were polished using a semiautomatic Buehler MiniMet 1000 setup (Buehler, Lake Bluff, IL, USA) to the mirror-like surface using 600, 800, and 1200 grit sandpapers and a suspension of silica nanoparticles as described elsewhere [23,24]. Finally, the disks were repeatedly cleaned with acetone and deionized water in an ultrasonic bath for 15 min.

The polished NiTi discs were placed in 100 mL glass vessels with dual-acid etchant (H_2_SO_4_/HCl/H_2_O *v*/*v*/*v* = 9/11/30, Vecton, Saint-Petersburg, Russia), basic piranha solution (NH_4_OH/H_2_O_2_ *v*/*v* = 7/3, Vecton, Saint-Petersburg, Russia), or acidic piranha solution (H_2_SO_4_/H_2_O_2_ *v*/*v* = 7/3, Vecton, Saint-Petersburg, Russia) for 30 and 120 min. Immediately after etching, the samples were washed in deionized water.

### 2.2. Samples Characterization

The surface morphology was studied with a scanning electron microscopy (SEM) (Zeiss Merlin) operated at 20 kV. The magnifications from 100 up to 400,000 at 3–5 random positions were used. The topography of the samples’ surfaces was tested by a Solver P47 Pro atomic force microscope (AFM) (NT-MDT, Moscow, Russia) using the tapping mode with 1 × 1 μm and 10 × 10 μm scan areas. For each sample, 5–7 random positions were probed. The vertical range (Rz), root mean square roughness (RMS), average roughness (Ra), and specific surface area (SA—the ratio of 3D surface area over 2D scan size, in percent) were calculated using the program Gwyddion 2.59.

The composition of the samples’ surfaces was determined by X-ray photoelectron spectroscopy (XPS) with an Escalab 250Xi spectrometer (Thermo Fisher Scientific, Waltham, MA, USA). For XPS measurements, samples were excited with Al Kα (1486.7 eV) X-rays, and high-resolution spectra were charge-compensated by setting the C1s carbon line binding energy at 284.8 eV.

The contact angle (CA) measurements were performed by the sessile drop method using a Theta Lite optical tensiometer (Biolin Scientific, Sweden). The optical tensiometer was equipped with OneAttension Software for obtaining and processing results of tensiometric measurements. The volume of liquid droplets did not exceed 2.2 µL. The mean CA value (Θ) for the same sample was obtained by averaging at least 12 results of repeated measurements. The surface free energy (SFE) was calculated by the Owens–Wendt–Rabel–Kaelble (OWRK)/Fowkes approach using the two-liquid method (water versus diiodomethane contact angles) [27]. Ultrapure water served as a liquid with a dominant polar component of SFE (SFE^p^ = 51.0 mN/m, SFE^d^= 21.8 mN/m), and diiodomethane stabilized by copper (99%, Sigma-Aldrich) was used as a liquid with a dominant dispersive component of SFE (SFE^d^ = 48.5 mN/m, SFE^p^ = 2.3 mN/m). The Cassie–Baxter equation [28] was used to account for the effect of surface roughness on the value of contact angles:

Cos Θr = r·Cos Θ_0_, (1)

where Θr and Θ_0_ are the contact angles for rough and flat surfaces, respectively, and r is the roughness factor (SA ratio), r > 1.0.

## 3. Results

### 3.1. Weight Loss, Morphology, and Topography

Regardless of the etchant type, etching for 30 min does not lead to a noticeable change in weight (Table 1), but after the 2-h process, a noticeable weight loss is observed for HCl/H_2_SO_4_ and NH_4_OH/H_2_O_2_ treatments. Samples etched in H_2_SO_4_/H_2_O_2_ for two hours reveal negligible weight loss (0.051%).

**Figure 1:**
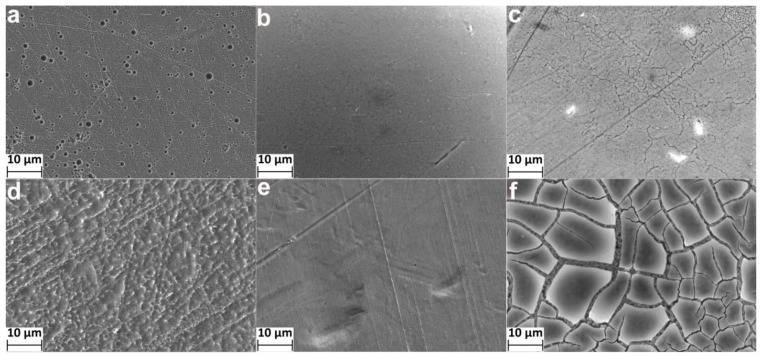
SEM images (10 µm scale bars) of nitinol surface for samples: (**a**) HCl/H_2_SO_4_-30 min, (**b**) H_2_SO_4_/H_2_O_2_-30 min, (**c**) NH_4_OH/H_2_O_2_-30 min, (**d**) HCl/H_2_SO_4_-120 min, (**e**) H_2_SO_4_/H_2_O_2_-120 min, (**f**) NH_4_OH/H_2_O_2_-120 min.

**Figure 2:**
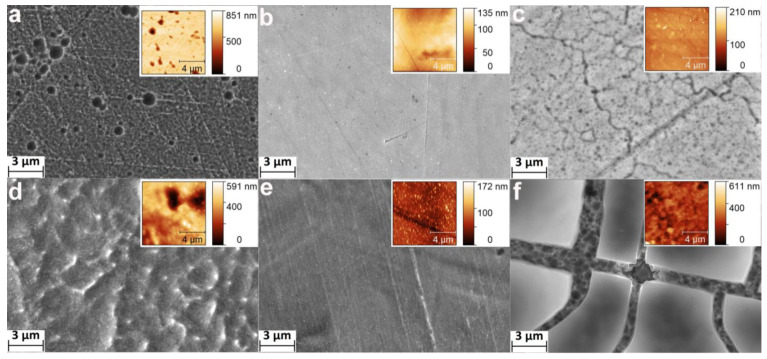
SEM images (3 µm scale bars) of nitinol surface for samples: (**a**) HCl/H_2_SO_4_-30 min, (**b**) H_2_SO_4_/H_2_O_2_-30 min, (**c**) NH_4_OH/H_2_O_2_-30 min, (**d**) HCl/H_2_SO_4_-120 min, (**e**) H_2_SO_4_/H_2_O_2_-120 min, (**f**) NH_4_OH/H_2_O_2_-120 min. The insets show the AFM surface topographies of the corresponding samples.

**Figure 3:**
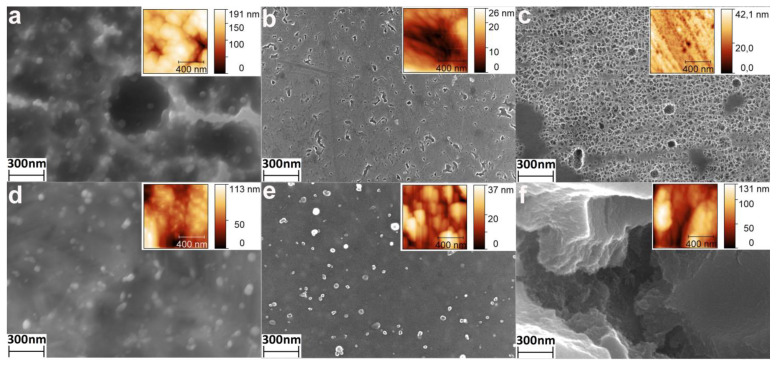
SEM images (300 nm scale bars) of nitinol surface for samples: (**a**) HCl/H_2_SO_4_-30 min, (**b**) H_2_SO_4_/H_2_O_2_-30 min, (**c**) NH_4_OH/H_2_O_2_-30 min, (**d**) HCl/H_2_SO_4_-120 min, (**e**) H_2_SO_4_/H_2_O_2_-120 min, (**f**) NH_4_OH/H_2_O_2_-120 min. The insets show the AFM surface topographies of the corresponding samples.

The microscale (Figure 1) submicron (Figure 2) and nanoscale (Figure 3) morphology of the etched samples were studied by SEM using different magnification. It was found that the HCl/H_2_SO_4_-30 min samples do not have a developed topography: the surface is slightly damaged, the scratches formed during grinding and polishing are still visible (Figure 1a, Figure 2a and Figure 3a). However, chaotically located micron-size pits resulting from surface erosion appeared. In addition, grains with a diameter of several tens of nanometers were observed (Figure 3a). With an increase in the etching time up to 2 h, the aforementioned pits disappeared (Figure 1d and Figure 2d), and a developed microscale topography with particles of a few tens of nanometers (Figure 3d) appeared.

Regardless of the process duration, the samples etched in H_2_SO_4_/H_2_O_2_ do not have a developed morphology (Figure 1b,e and Figure 2b,e)and did not differ from the unetched polished NiTi (Figure S1). However, nanosized pits (Figure 3b) and nanoparticles (Figure 3e) were observed on the surface.

The morphology of the samples etched in NH_4_OH/H_2_O_2_ changed significantly. After etching for 30 min, a nanoscale porous structure and cracks were formed (Figure 2c and Figure 3c). The surface of the samples etched during 120 min consisted of relatively flat areas (Figure 1f) no more than 20–30 microns in size, separated by deep cracks (Figure 2f and Figure 3f).

The topography of the samples was studied by AFM using 10 × 10 (Figure 2, Insets) and 1 × 1 µm (Figure 3, Insets) scanning areas. AFM 3D surface topographies showed the same surface features (pits, particles, cracks, etc.) as the SEM images.

**Figure 4:**
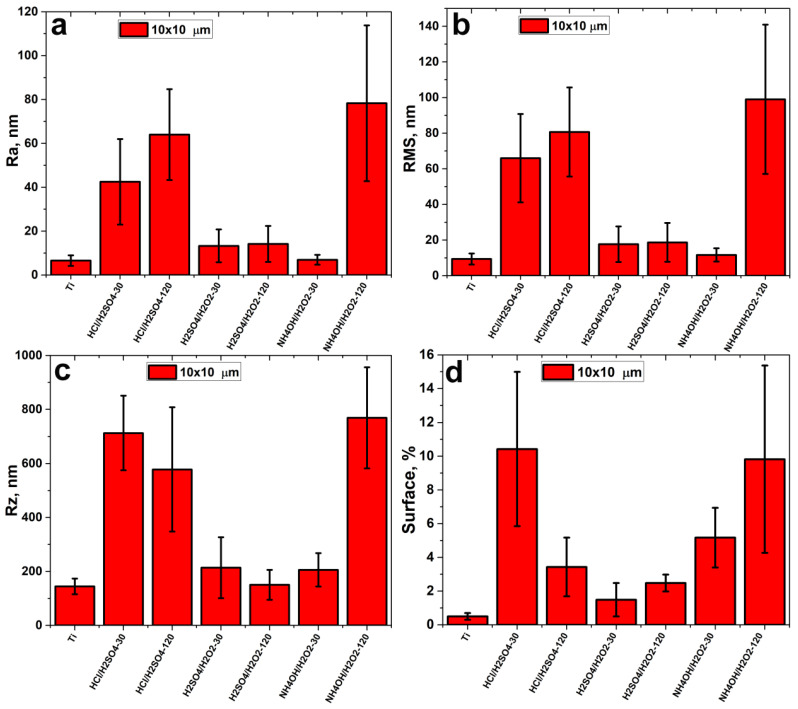
AFM topography parameters for etched nitinol samples: average roughness (Ra)—(**a**), root mean square roughness (RMS)—(**b**), the vertical range (Rz)—(**c**), and specific surface area—(**d**).

Surface topography parameters such as average roughness (Ra—Figure 4a), root mean square roughness (RMS—Figure 4b), the vertical range (Rz—Figure 4c), and specific surface area (SSA—ratio of 3D surface area to 2D scan size—Figure 4a) were calculated using AFM data. Etching in H_2_SO_4_/H_2_O_2_ slightly increased Ra, Rz, RMS, and SA. Etching in HCl/H_2_SO_4_ and NH_4_OH/H_2_O_2_-120 min significantly increased roughness, height difference and surface area. Topography parameters for NH_4_OH/H_2_O_2_-120 min and HCl/H_2_SO_4_ samples are very close to each other, but the real Ra, RMS, Rz, and surface area for NH_4_OH/H_2_O_2_-120 min sample should be greater because it has very deep cracks which cannot be properly measured by AFM. It is noteworthy that, for the NH_4_OH/H_2_O_2_-30 min sample, the roughness and maximum height difference practically did not increase (Figure 4c), but the specific surface area did increase (Figure 4d).

### 3.2. Surface Composition and Wettability

**Figure 5:**
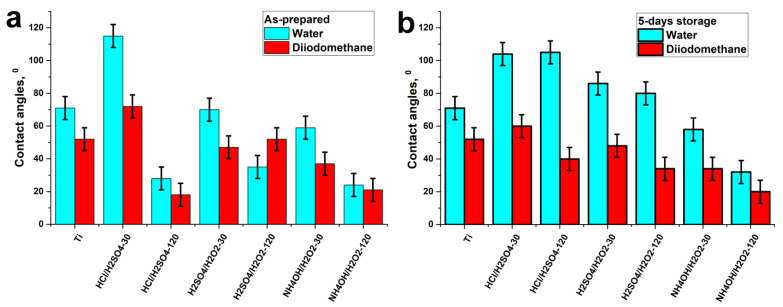
Results of water and diiodomethane contact angle measurements of as-prepared (**a**) and aged in the air (**b**) samples.

The wettability of samples strongly depends on the type of etchant and the duration of etching (Figure 5). The initial surface of nitinol is hydrophilic (CA = 71 ± 7°). Measurement of the water contact angles for the surface of as-prepared samples (immediately after etching within 1 h) showed that the treatment with HCl/H_2_SO_4_-30 min increased the water contact angle (WCA), and the surface became hydrophobic (Figure 5a). However, the longer the etching, the more hydrophilic the surface became (28 ± 7°). In general, a long-term etching (120 min) results in a more hydrophilic surface than a short one (30 min) for all etchants used. It is noteworthy that with aging (stored in the air), an increase in WCAs is observed for all samples except for HCl/H_2_SO_4_-30 min and NH_4_OH/H_2_O_2_-120 min (Figure 5b). Only NH_4_OH/H_2_O_2_ samples stored for 5 days in the air remained hydrophilic.

**Figure 6:**
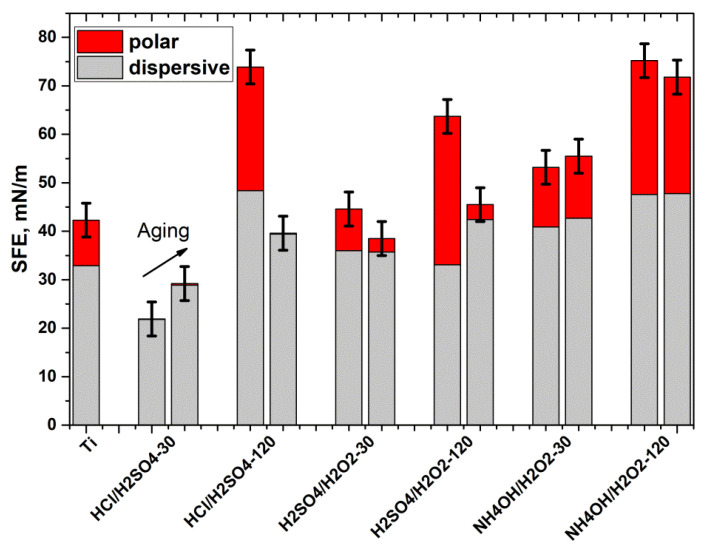
Results of the surface free energy calculation for unetched and etched nitinol.

Analysis of the calculated surface free energy values showed that the hydrophilicity of the samples is due to the polar component (Figure 6). The hydrophobic HCl/H_2_SO_4_-30 min sample has practically no polar component, and all SFE is due to the dispersive component. Hydrophilic freshly prepared HCl/H_2_SO_4_-120 min and H_2_SO_4_/H_2_O_2_-120 min samples had a significant polar component (Figure 6), but they lost it with aging and became more hydrophobic (Figure 5b). Only the hydrophilicity of NH_4_OH/H_2_O_2_ samples was stable during aging: their WCA values (Figure 5b) and SFE values (Figure 6) did not change significantly.

The chemical composition of the surface of the samples was investigated by XPS. In addition to nickel, titanium, and oxygen, carbon was detected on the surface of the samples, as well as sulfur for the samples etched in HCl/H_2_SO_4_ and H_2_SO_4_/H_2_O_2_, and nitrogen for all NH_4_OH/H_2_O_2_ samples. The quantitative analysis showed that the surface layer of unetched NiTi is enriched in titanium with a ratio Ti/Ni = 4.1 (Table 2). Upon etching in acidic piranha solutions, this ratio increased, reaching 32.7 for the H_2_SO_4_/H_2_O_2_-30 min sample. SEM energy dispersive X-ray (EDX) analysis showed that Ni and Ti were evenly distributed over the material (Figures S2 and S3). When etching NH_4_OH/H_2_O_2_, the ratio decreased significantly because titanium was mostly etched out and the surface was greatly enriched with nickel (Ti/Ni = 0.14–0.16). SEM-EDX measurements (Figures S4 and S5) showed that a more oxidized and nickel-enriched surface is typical for micro-sized plates, and deep cracks contain nickel and titanium in a nearly equiatomic ratio.

**Figure 7:**
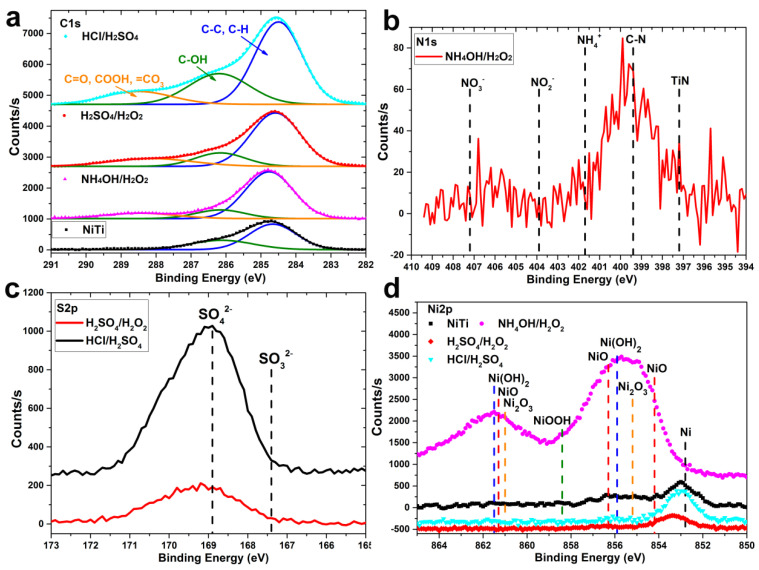
High-resolution XPS spectra of polished NiTi and etched samples: C1s (**a**), N1s (**b**), S2p (**c**), Ni2p (**d**).

In the high-resolution C1s spectra, two intense peaks at 284.8 eV and 286.3 eV were observed for all samples (Figure 7a). These peaks correspond to aliphatic hydrocarbons (C-C, C-H) and hydroxyl (C-OH) groups, respectively. Moreover, a small shoulder at higher binding energies (287–290 eV), assigned to carboxyl and aldehyde groups, was observed for the etched samples. All these carbon-containing species are originated from the adsorption of organic contaminations from the air during the sample storage.

For the samples etched in NH_4_OH/H_2_O_2_, a small amount of nitrogen was observed on the surface. The weak peaks of the N1s’ spectrum (Figure 7b) correspond to the C-N bond, but not to the NH_4_^+^ and NO_3_^−^ or NO_2_^−^ species. Therefore, we assume that the presence of nitrogen is caused by external contamination and not the result of etching in a nitrogen-containing etchant. On the other hand, sulfur was detected on the surface of the HCl/H_2_SO_4_ and H_2_SO_4_/H_2_O samples probably as a result of chemical etching in solutions containing H_2_SO_4_. The BE peak maxima in the S2p spectra correspond to SO_4_^2−^ group (Figure 7c).

Analysis of the Ni2p spectra showed (Figure 7b) that the surfaces of the unetched NiTi and NiTi etched in HCl/H_2_SO_4_ and H_2_SO_4_/H_2_O_2_ contain nickel predominantly in the metallic state, and etching in NH_4_OH/H_2_O_2_ leads to nickel oxidation with the formation of oxides and hydroxides.

**Figure 8:**
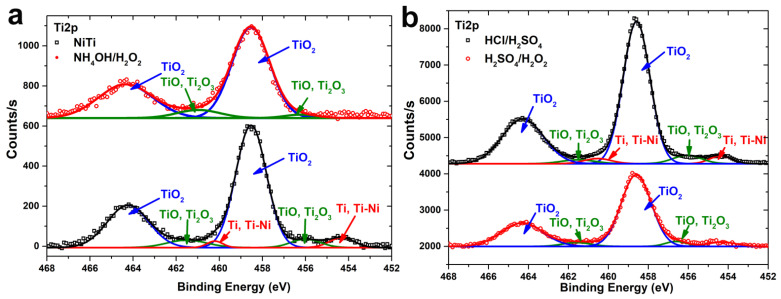
High-resolution Ti2p XPS spectra of polished NiTi and etched in NH_4_OH/H_2_O_2_-120 min samples (**a**) and etched in HCl/H_2_SO_4_-120 min and H_2_SO_4_/H_2_O_2_-120 min (**b**).

According to high resolution Ti2p XPS spectra (Figure 8), the Ti 2p_3/2_ and Ti 2p_1/2_ peaks are located at 458.6 eV and 464.3 eV respectively for all samples and can be attributed to Ti^4+^ (Ti 2p_1/2_–Ti 2p_3/2_ splitting equal to 5.7) [29]. Low-intensity Ti^3+^ or Ti^2+^ shoulders at lower binding energies were detected for all samples. Metallic Ti or Ti–Ni peaks at 454.3 and 460.3 eV are quite evident in the spectrum of polished NiTi (Figure 8a) and HCl/H_2_SO_4_ (Figure 8b) samples.

**Figure 9:**
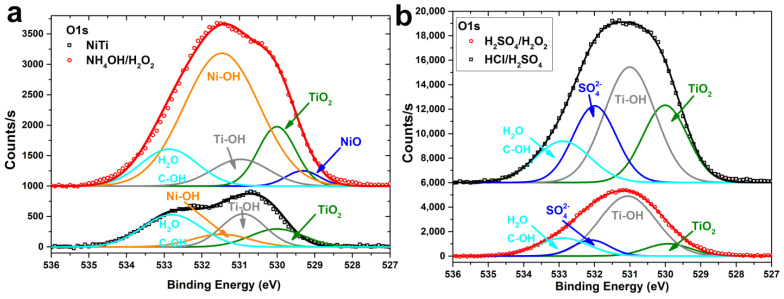
High-resolution O1s XPS spectra of polished NiTi and etched in NH_4_OH/H_2_O_2_-120 min samples (**a**) and etched in HCl/H_2_SO_4_-120 min and H_2_SO_4_/H_2_O_2_-120 min (**b**).

O1s spectra showed the presence of TiO_2_, Ti–OH, H_2_O/C-OH groups for all samples (Figure 9). Moreover, for NiTi and NH_4_OH/H_2_O_2_ samples (Figure 9a), Ni–OH peaks are observed, and, for NH_4_OH/H_2_O_2,_ a less intense NiO peak is also seen. For HCl/H_2_SO_4_ and H_2_SO_4_/H_2_O_2_ samples, SO_4_^2−^ peaks are present at 532.1 eV (Figure 9b).

## 4. Discussion

### 4.1. Surface Morphology and Topography

Wet chemical etching of metals is a very complex process. It includes many physical and chemical stages such as material removal, surface functionalization, oxidation and passivation. These steps depend on the composition of the etchant and etching conditions (time, temperature, mixing, etc.). Depending on the conditions, the etching process obeys either diffusion or kinetic control. In diffusion control, the process rate is determined by the rates of reagent supply and reaction product removal from the surface. As a result, the etching proceeds according to the layer-by-layer mechanism, and the roughness of the resulting surface is smoothed. The limiting step of the kinetic control etching is the chemical reaction on the local areas of the surface. In the case of surface heterogeneity and meaningful difference in the reactivity of local areas, the surface topography changes significantly.

It is important to note that the surfaces of the NiTi samples were mechanically treated (polished) before etching. Therefore, their surfaces were initially modified. We believe that the mirror-like surfaces of polished NiTis were homogenous. In this case, uniform etching of such surfaces using a layer-by-layer material removal mechanism is assumed. In this work, this type of etching is observed for the H_2_SO_4_/H_2_O_2_ mixture. Slow weight loss was observed (Table 1), but no significant changes of morphology and topography of the surface occurred (Figure 1, Figure 2, Figure 3 and Figure 4). Only nanoscale pits and particles were formed for the H_2_SO_4_/H_2_O_2_-30 min and H_2_SO_4_/H_2_O_2_-120 min samples, respectively. It was previously shown [23,30] that a noticeable change in the morphology and topography of the pure Ti surface upon etching in H_2_SO_4_/H_2_O_2_ begins to occur from 1–2 h from the start of etching. Probably, the NiTi sample requires a longer etching to form a developed morphology and topography than the Ti sample due to differences in thickness and/or composition of the surface layer. For HCl/H_2_SO_4_ and NH_4_OH/H_2_O_2_ etching, the weight loss within 30 min is also insignificant. At the same time, the formation of micro-sized pits (pitting etching) and nanosized grains was observed for the HCl/H_2_SO_4_-30 min samples, as well as microsized cracks with a “spongy” nanostructure for the NH_4_OH/H_2_O_2_-30 min samples. It can be assumed that these very active etchants can quickly remove the surface layer, and the etching process occurs by the mechanism of kinetic control with an inhomogeneous surface. We can also conclude that etching will proceed similarly in the case of an untreated additive manufactured nitinol surface. At longer etching (2 h), the developed microsized topography and high surface roughness were revealed. On the surface of the NH_4_OH/H_2_O_2_-120 min sample, flat plates of no more than 10–20 µm in size are formed, separated by deep microscale cracks. We believe that cracks are formed in regions with more defects and therefore thermodynamically more active for chemical etching grain boundaries. The formation of pits and different nanoscale and microscale structures can be explained by the selective etching of structure/composition defects of grains, as well as local heterogeneous areas on the solid–liquid interface.

Based on sample thickness and relative weight loss, wet chemical etching removes a very thin layer from several hundred nanometers (H_2_SO_4_/H_2_O_2_ and HCl/H_2_SO_4_-30 min etching) to several micrometers (NH_4_OH/H_2_O_2_ and HCl/H_2_SO_4_-120 min). Therefore, it can be assumed that the homogeneous surface layer that appears during polishing is very thin.

It should be noted that AFM images of surface topography converge with SEM images (Figure 2 and Figure 3). However, analysis of AFM data allows quantification of the surface topography. The task of comprehensive quantification of topography using AFM data is rather complicated [4,31], so we calculated the different topography parameters such as arithmetic mean and root means square roughness, maximum height amplitudes, and specific surface area. Roughnesses values are robust parameters of the overall topography of etched surfaces, but maximum height is very sensitive to noises, defects, pits, and spikes; and surface area indicates the degree of the surface topography development [4]. H_2_SO_4_/H_2_O_2_ etching did not significantly change any of the AFM parameters, but HCl/H_2_SO_4_ changed them significantly (Figure 4). It is noteworthy that etching in HCl/H_2_SO_4_ for 30 min strongly increased the maximum height and surface area, while the same etching for 120 min increased the surface roughness. This difference is caused by the formation of deep pits during 30 min-etching, and the increase in the roughness of HCl/H_2_SO_4_-120 min samples is a result of the formation of a more developed topography. Notably, etching in NH_4_OH/H_2_O_2_ for 30 min practically did not alter the roughness and maximum height difference (Figure 4c) but increased the specific surface area (Figure 4d) due to the nanoscale porous structure (Figure 3c). Etching in NH_4_OH/H_2_O_2_ for 120 min significantly increased all topographic parameters, and the resulting topography is the most developed of all.

### 4.2. Surface Energy and Composition

In addition to material removal, surface passivation and oxidation can occur during wet chemical etching. Based on the XPS data, the oxide layer on the surface of polished NiTi is very thin, since metallic Ti is seen in the Ti2p spectra (Figure 8a). After etching in HCl/H_2_SO_4_, this peak is also clearly seen, but after H_2_SO_4_/H_2_O_2_ and NH_4_OH/H_2_O_2_ etching, it is absent, that indicates the surface oxidation, i.e., the surface oxide layer becomes so thick that the basic NiTi alloy is not detected by the XPS method. Moreover, it should be noted that the oxidation process continues even after etching when the samples are stored in air. This is evidenced by the change in surface energy with aging. The surfaces of the as-prepared samples have a high polar component of the surface free energy, which can be explained by the presence of a very thin oxide layer. However, when samples are stored in the air, the SFE diminished due to the disappearance of its polar component. In the case of NH_4_OH/H_2_O_2_, the oxide-hydroxide surface layer is rather thick for as-prepared samples, and the polar component is due to different surface composition, which remains unchanged when stored in air.

There are currently insufficient data on the chemical etching of NiTi. Previously, chemical etching has only been used as a tool to solve various purposes but has not been studied in detail [16,17,18,19,20,21]. Possible reactions and mechanisms of etching and the formation of various micro- and nanoscale structures and surface composition variations were not discussed there. Nevertheless, the data obtained in this work and available from the literature allow us to make some assumptions and conclusions.

The chemistry of NiTi etching is quite complex. Various chemical reactions can take place in which both titanium and nickel are involved. In the case of H_2_O_2_-containing solutions, both simple oxidation to TiO_2_, Ti_2_O_3_, TiO [32] and the formation of hydroxo and peroxo complexes of titanium (TiO(OH)_2_, Ti(H_2_O_2_)O_2_) can occur [33,34]. Etching with H_2_SO_4_/H_2_O_2_ and HCl/H_2_SO_4_ also produces Ti(SO_4_)_2_ and TiOSO_4_ surface compounds. It should be noted that no chlorides were detected when HCl/H_2_SO_4_ was used. The NH_4_OH/H_2_O_2_ etching probably results in fewer titanium peroxocomplexes, but more soluble and easily formed ammonia complexes such as [Ti(NH_3_·H_2_O)_2_]^4+^ [25]. As a result, the titanium can be removed into solution and excess nickel remains on the surface as an oxide-hydroxide (NiO, Ni_2_O_3_, Ni(OH)_2_). According to XPS data, the surfaces of all samples contain Ti–OH species, but it is unlikely that it is titanium hydroxide, since these groups were found even after etching in strong acidic mixtures—HCl/H_2_SO_4_. The titanium is probably hydroxylated when the samples are washed in water after etching.

One of the most interesting obtained results is the change in ratio Ni/Ti after etching. We assume that the enrichment of the surface with titanium is caused by the dissolution of nickel with the simultaneous oxidation of titanium and the growth of the surface oxide layer (TiO_2_ or TiO_x_). Titanium is more readily oxidized than a nickel from a thermodynamic point of view:

Ti(s) + O_2_(g) → TiO_2_(s)  ΔHf = −956 kJ/mol (2)

Ni(s) + 1/2O_2_(g) → NiO(s)  ΔHf = −241 kJ/mol (3)

The large disparity between the enthalpy of the TiO_2_ and NiO may reflect the stronger affinity of Ti than that of Ni for oxidation. Such a type of oxidation could be realized when H_2_O_2_ is used. According to XPS data, these processes are observed during etching in H_2_SO_4_/H_2_O_2_ and partly in HCl/H_2_SO_4_. Under these conditions, the thickness of the titanium oxide layer increases, and the nickel is present on the surface in a small amount in the metallic state. After etching in NH_4_OH/H_2_O_2_, the surface is enriched with nickel. Apparently, in this case, nickel is oxidized under the action of H_2_O_2_ to an insoluble oxide NiO_x_ (Figure 7d), and titanium dissolves and goes into solution in the form of ammine complexes such as Ti(NH_3_)_x_^4+^ or TiO(NH_3_)_x_^2^+. We believe that titanium dissolves precisely under the action of NH_4_OH, since according to literature when exposed to H_2_O_2_, the nitinol surface should be enriched with titanium due to titania formation [35].

### 4.3. Prospects for NiTi Chemical Etching in Medicine

As mentioned in the Introduction section, the HCl/H_2_SO_4_ mixture is actively used in SLA technology to modify the surface of titanium implants. The HCl/H_2_SO_4_ etching agent improves surface topography and successfully removes SiO_2_ and Al_2_O_3_ particles that remain on the implant surface after sandblasting. Based on a large number of in vitro and in vivo studies, researchers agree that developed morphology and topography at the macro-, micro- and nanoscale are vital for the most successful biointegration of the material [4,12,31,36]. From a biomechanical point of view, macro-roughness improves the implant surface area as well as increases the friction coefficient and the implant stability. Microscale structures, especially micropores and micropits, significantly enhance the adhesion of bone tissue cells. Specific nano-roughness and nanostructures are very important parameters to improve circulation, adsorption and increase the number of sites for various biomolecules (signaling proteins, cytokines, integrins, hormones, and growth factors, etc.) and nutrients. Moreover, the nanostructures have an antibacterial effect [37].

The results of this study showed that the HCl/H_2_SO_4_ treatment forms a developed morphology and topography of the nitinol surface. The NH_4_OH/H_2_O_2_ mixture is an equally successful etchant for NiTi, and the 2 h-treatment results in deep cracks on the surface, separating flat plates no larger than 10–20 µm in size. Such surface morphology has great prospects for use in medicine, because the size of the plates is close to the size of cells such as osteoblasts, fibroblasts, mesenchymal cells, etc., and deep cracks can promote the formation and growth of cell appendages [36].

The surface composition is also very important for successful medical applications. The most valuable parameter is the presence of a large concentration of hydrophilic surface species [38]. This leads to faster adsorption of different biomolecules and affects cell activity [39]. From this point of view, the advantages of the NH_4_OH/H_2_O_2_ etching are obvious. Another important parameter is the presence of a rather thick layer of biocompatible titanium oxide, which prevents biocorrosion and diffusion of metallic Ni. Although nitinol is considered a corrosion-resistant alloy, the nickel in it is extremely toxic and carcinogenic [40,41]. From this point of view, the samples etched with NH_4_OH/H_2_O_2_ are much worse than those etched with HCl/H_2_SO_4_ and even H_2_SO_4_/H_2_O_2_, since their surfaces are nickel-enriched. Nevertheless, the disadvantages of the surface composition produced by chemical etching can be compensated by additional surface modification allowing to preserve the morphology and topography of the surface.

To date, the in vitro results on chemically etched NiTi have been presented only in two works [10,19], while the in vivo results are completely absent. Two-stage chemical etching in HCl/HF/H_3_PO_4_ and HNO_3_/HCl mixtures was shown to be promising for significantly improving the adhesion and proliferation of MG-63 preosteoblast cells. Yamasaki et al. showed that NiTi anodized in HNO_3_/H_3_PO_4_ solutions enhanced the attachment and spreading MC3T3-E1 osteoblast-like cells regardless of the etching mixture ratio [10]. In this regard, our future plans are to study the effect of chemical etching of nitinol in HCl/H_2_SO_4_, H_2_SO_4_/H_2_O_2,_ and NH_4_OH/H_2_O_2_ on the in vitro as well as in vivo response of various cells.

## 5. Conclusions

A comparative study of NiTi etching showed that H_2_SO_4_/H_2_O_2_ does not significantly change the surface morphology and roughness, whereas etching in HCl/H_2_SO_4_ leads to the formation of a developed microsized topography and nanograins. Prolonged etching (2 h) in an H_2_SO_4_/H_2_O_2_ or HCl/H_2_SO_4_ mixture significantly increases the hydrophilicity, as indicated by an increase in the polar component of surface free energy, but hydrophilicity drops when samples are stored in air for 5 days. All samples etched in mixtures containing H_2_SO_4_ showed the presence of a small amount of SO_4_^2−^ groups on the surface. Etching in NH_4_OH/H_2_O_2_ dissolves titanium and oxidizes nickel to form its oxide and hydroxide, and steadily increases hydrophilicity over time, as indicated by the increase in surface energy mainly due to its polar component. The surface morphology and topography of the samples etched in NH_4_OH/H_2_O_2_ strongly depend on the duration of etching. At a 30-min etching exposure, the surface is characterized by a spongy nanostructure with low roughness but a large surface. Etching for 2 h leads to the formation of deep microscale cracks separating flat plates not more than 10–20 µm in size. The results presented will allow us to develop an approach for creating surfaces with physicochemical properties that can be varied over a wide range, including for optimization for specific applications.

## Figures and Tables

**Table 1:** Weight loss of chemically etched nitinol.

Type of Chemical Etching	Weight before CE, g	Weight after CE, g	Weight Loss, g	Weight Loss, %
HCl/H_2_SO_4_-30 min	1.71360	1.71324	0.00036	0.021
HCl/H_2_SO_4_-120 min	1.78714	1.78389	0.00325	0.182
H_2_SO_4_/H_2_O_2_-30 min	1.52844	1.52787	0.00057	0.037
H_2_SO_4_/H_2_O_2_-120 min	1.62349	1.62267	0.00083	0.051
NH_4_OH/H_2_O_2_-30 min	1.32186	1.32125	0.00061	0.046
NH_4_OH/H_2_O_2_-120 min	1.43172	1.42771	0.00402	0.281

**Table 2:** Data of XPS quantitative analysis.

Sample	Ti2p	Ni2p	O1s	C1s	N1s	S2p
NiTi	8.29	2.01	29.75	59.95	-	-
HCl/H_2_SO_4_-30 min	6.18	0.46	37.72	53.15	-	2.49
HCl/H_2_SO_4_-120 min	10.20	0.47	44.89	41.70	-	2.74
H_2_SO_4_/H_2_O_2_-30 min	8.83	0.27	40.62	48.56	-	1.72
H_2_SO_4_/H_2_O_2_-120 min	6.56	0.52	36.50	54.79	-	1.64
NH_4_OH/H_2_O_2_-30 min	2.05	14.24	43.95	37.68	2.08	-
NH_4_OH/H_2_O_2_-120 min	2.95	17.89	36.92	40.47	1.76	-

## Data Availability

The main data had been provided in the paper and Supplementary Material. Any other raw/processed data required to reproduce the findings of this study are available from the corresponding author upon request.

## Supplementary Materials

The following are available online at https://www.mdpi.com/article/10.3390/ma14247683/s1, Figure S1: SEM images of polished NiTi at different magnifications—200,000× (a), 100,000× (b), 10,000× (c) 3000× (d). In the insets—AFM surface topographies. Figure S2. SEM images of NiTi etched in H_2_SO_4_/H_2_O_2_-30 min and the results of EDX chemical analysis. Figure S3. SEM images of NiTi etched in H_2_SO_4_/H_2_O_2_-120 min and the results of EDX chemical analysis. Figure S4. SEM images of NiTi etched in NH_4_OH/H_2_O_2_-30 min and the results of EDX chemical analysis. Figure S5. SEM images of NiTi etched in NH_4_OH/H_2_O_2_-120 min and the results of EDX chemical analysis.
