# Supplementary material for: Surface Modification of Additively Manufactured Nitinol by Wet Chemical Etching
Source: Materials (Basel). 2021 Dec 13;14(24):7683. doi: 10.3390/ma14247683 (PMC8708015; doi:10.3390/ma14247683)
Supplement: Supplementary file 1 [file materials-14-07683-s001.zip › materials-1491440-supplementary.pdf]

Surface Modification of Additively Manufactured Nitinol by Wet Chemical Etching

Denis Nazarov [1,2,*], Aida Rudakova [3], Evgenii Borisov [1] and Anatoliy Popovich [1]

1 Peter the Great Saint Petersburg Polytechnic University, Polytechnicheskaya, 29, 195221 Saint Petersburg, Russia; evgenii.v.borisov@gmail.com (E.B.); director@immet.spbstu.ru (A.P.)
2 Research Centre "Innovative Technologies of Composite Nanomaterials", Saint Petersburg State University, Universitetskaya Nab, 7/9, 199034 Saint Petersburg, Russia
3 Laboratory "Photoactive Nanocomposite Materials", Saint Petersburg State University, Ulianovskaia str. 1, Peterhof, 198504 Saint Petersburg, Russia; aida.rudakova@spbu.ru
* Correspondence: dennazar1@yandex.ru

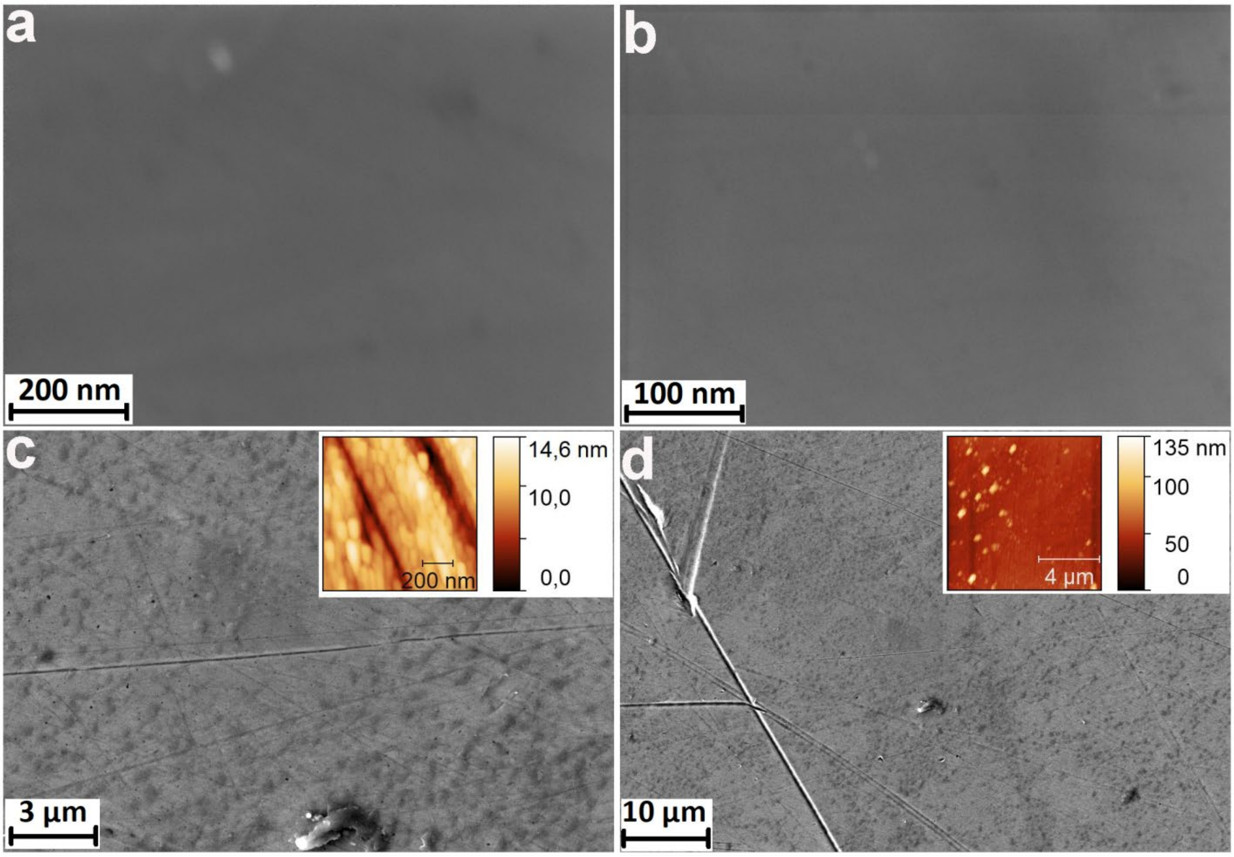

Figure S1. SEM images of polished NiTi at different magnifications – 200 000x (a), 100 000x (b), 10 000x (c) 3000x (d). In the insets - AFM surface topographies.

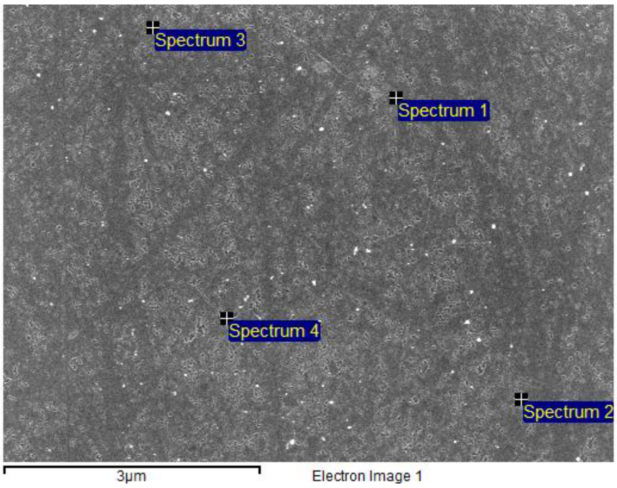

Spectrum	C	Ti	Ni
Spectrum 1	40.00	30.27	29.73
Spectrum 2	22.01	39.22	38.77
Spectrum 3	28.35	36.30	35.35
Spectrum 4	26.19	37.21	36.60
Mean	29.13	35.75	35.11
Std. deviation	7.70	3.85	3.86
Max.	40.00	39.22	38.77
Min.	22.01	30.27	29.73

Figure S2. SEM images of NiTi etched in H_2SO_4/H_2O_2-30min and the results of EDX chemical analysis.

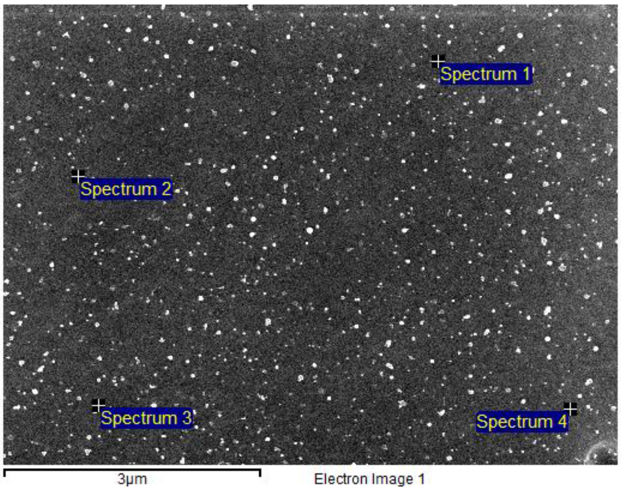

Spectrum	Ti	Ni
Spectrum 1	49.59	50.41
Spectrum 2	48.48	51.52
Spectrum 3	49.60	50.40
Spectrum 4	49.53	50.47
Mean	49.30	50.70
Std. deviation	0.55	0.55
Max.	49.60	51.52
Min.	48.48	50.40

Figure S3. SEM images of NiTi etched in H_2SO_4/H_2O_2-120min and the results of EDX chemical analysis.

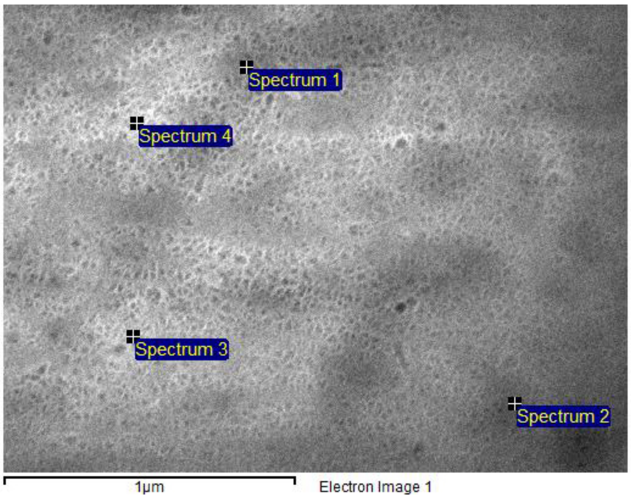

Spectrum	O	Ti	Ni
Spectrum 1	39.10	28.98	31.92
Spectrum 2	38.52	29.33	32.14
Spectrum 3	38.26	29.27	32.47
Spectrum 4	40.82	27.48	31.70
Mean	39.17	28.77	32.06
Std. deviation	1.15	0.87	0.33
Max.	40.82	29.33	32.47
Min.	38.26	27.48	31.70

Figure S4. SEM images of NiTi etched in NH_4OH/H_2O_2-30min and the results of EDX chemical analysis.

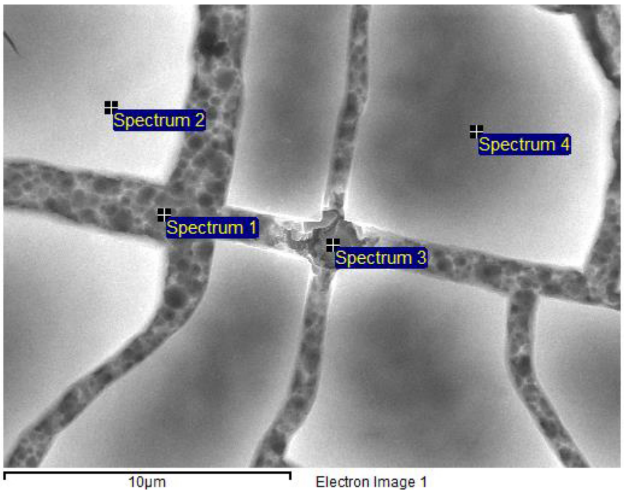

Spectrum	O	Ti	Ni
Spectrum 1	27.12	34.84	38.03
Spectrum 2	67.84	10.93	21.24
Spectrum 3	30.06	28.87	41.06
Spectrum 4	67.41	12.40	20.19
Mean	48.11	21.76	30.13
Std. deviation	22.57	11.93	10.95
Max.	67.84	34.84	41.06
Min.	27.12	10.93	20.19

Figure S5. SEM images of NiTi etched in NH_4OH/H_2O_2-120min and the results of EDX chemical analysis.
